# Topical Allopurinol-Loaded Nanostructured Lipid Carriers: A Novel Approach for Wound Healing Management

**DOI:** 10.3390/bioengineering8120192

**Published:** 2021-11-28

**Authors:** Carla Varrica, Manuela Carvalheiro, Catarina Faria-Silva, Carla Eleutério, Giuseppina Sandri, Sandra Simões

**Affiliations:** 1 Department of Drug Sciences, University of Pavia, 27100 Pavia, Italy; carlagrazia.varrica01@universitadipavia.it (C.V.); g.sandri@unipv.it (G.S.); 2 Faculty of Pharmacy, Universidade de Lisboa, 1649-003 Lisboa, Portugal; mcarvalheiro@ff.ulisboa.pt (M.C.); anacatarinafs@hotmail.com (C.F.-S.); carla.vania@campus.ul.pt (C.E.)

**Keywords:** wound healing, allopurinol, topical formulations, nanocarrier systems, nanostructured lipid carriers

## Abstract

Nanostructured lipid carriers (NLC) have been widely studied as delivery systems for a variety of routes, including the skin. Their composition results in an imperfect lipid matrix, allowing increased drug encapsulation. Allopurinol (AP), a xanthine oxidase inhibitor, is characterized by low water solubility and high melting point, which has hampered its use through the topical route. In this work, AP was incorporated in a NLC formulation to enhance drug-carrier association and skin delivery as a topical approach to treat wounds. AP-NLC system was characterized in terms of size, charge, rheological behavior, and in vitro skin permeation. The in vitro cytotoxicity was evaluated using HaCaT cells. The wound healing efficacy of the AP-NLC formulation on animal skin lesions was evaluated in male Wistar rats. The AP-NLC presented a mean size of 193 ± 15 nm with a PdI of 0.240 ± 0.02, zeta potential values around −49.6 mV, and an encapsulation efficiency of 52.2%. The AP-NLC formulation presented an adequate profile to be used topically, since epidermal and dermal drug retention were achieved. No reduction in HaCaT cells viability was observed at the tested concentrations (AP < 10 μg/mL). The in vivo application of the AP-NLC formulation resulted in the regeneration of skin lesions when compared with non-treated controls.

## 1. Introduction

Wound healing management has been extensively studied, since currently available approaches do not completely respond to the need of reducing the incidence of nonhealing wounds [1,2]. A successful healing system should be capable of accelerating the wound closure, reducing infection, and stimulating healing mechanisms, mimicking the extracellular matrix feature, moisture the wound, and reducing scar formation [3]. The development of effective novel wound therapies to better manage patients with chronic wounds is an intense area of research to identify the physiological and metabolic key players in wounds’ prolonged inflammatory phase. In recent years, studies have revealed that uric acid is elevated in wound fluid, and that higher concentrations are correlated with increased wound severity [4,5]. The detrimental role of xanthine oxidoreductase (XOR) in impaired healing role of in wounds is gaining increased interest, as XOR expression and activity are upregulated in the chronic wound environment [6]. These events, once associated with the overproduction of reactive oxygen species (ROS), result in prolonged closure and sustained inflammation [6]. Thus, targeting wound XOR may offer a mode to control local ROS, possibly achieved through topical application of XOR inhibitors. The idea of treating wounds with topical allopurinol (AP) decreasing the uric acid and ROS released into the wound environment has been already hypothesized [4]. AP is a molecule with a structure very similar to hypoxanthine, and this similarity allows the drug to inhibit the enzymes with hypoxanthine as a substrate. This exemplary xanthine oxidase inhibitor has been the keystone for the clinical management of gout and conditions associated with hyperuricemia for several decades [7]. Additionally, this drug is inexpensive and can be easily monitored via its breakdown product oxypurinol. However, AP is characterized by low solubility in water and a high melting point, which has restricted its use only to the oral route. The only topical AP study reported so far shows that AP (30 µg) topically applied on excisional wounds made on male C57BL/ 6 mice significantly impaired wound healing [8]. However, authors confirmed the xanthine oxidase abundance in wounds.

Topical treatments represent the classic approach to wound management. This approach uses colloidal agents, antiseptics, and antibacterials to prevent infections [3]. Concretely, and with the exception of Madigan and co-workers’ study [8], there are only few studies regarding AP topical administration for wound management. The indication of an AP topical form has been described for the treatment of hand-foot syndrome (HFS), in a dose-limiting toxicity of capecitabine on a trial conducted on the basis of preliminary data that a 3% allopurinol-based topical agent may prevent HFS [9,10]. Topical AP was as effective as established drugs, namely steroids and acetylcysteine, in the early treatment of experimental alkali corneal burns [11]. A case report shows that a tattoo skin reaction was possible to treat with 3% allopurinol oil-in-water anionic emulsion, topically, three times a day for three months [12].

In fact, the topical route represents an advantageous alternative route, as it allows a localized treatment without an involvement of the systemic route, promotes controlled drug delivery, and avoids first-pass metabolism, reducing the toxicity associated with the drug [13]. Moreover, the drug can be exposed only to affected skin, allows for self-administration and, consequently, determines good patient compliance [14]. Several factors influence the efficacy of the drug in topical administration: pharmacokinetics aspects, physico-chemical properties, and the interaction with the skin [13]. Nanostructured biomaterials, such as lipid-based nanocarriers, present good physiochemical properties for the delivery of several drugs, overcoming some of the limitations of conventional therapies [15]. Topical formulations can be applied when the skin is defective, such as in the case of wounds, or when the skin barrier is intact. In the first case, the percutaneous absorption is increased, since the permeability of the skin is highly variable in comparison to normal skin, and results in higher absorption of the drug, especially hydrophilic molecules [16]. In the case of an intact barrier, the formulation should promote deep skin drug delivery. In the case of wounds, the topical delivery may have different targets as the drug needs to be delivered in the *stratum corneum* (SC, the outermost layer of epidermis), epidermis, or dermis. Furthermore, the vehicle should guarantee the drug delivery and promote the drug retention within the skin [16]. Many encouraging results achieved by the topical treatment of wounds using new topical delivery system have opened a stimulating nanotechnology area of research and for this reason several lipid-based formulations are involved in clinical trials [17].

Due to its physico-chemical characteristics, the formulation of topical AP products may constitute a technological challenge. Nevertheless, the encapsulation of AP has been performed using different nanocarrier types for different therapeutic purposes. AP-loaded poly (ethyl cyanoacrylate) nanoparticles were developed and successfully tested against *Trypanosoma cruzi* in comparison to the free drug [18]; AP was encapsulated into bovine serum albumin nanoparticles for kidney targeting of the drug [19], or loaded into chitosan nanoparticles for sustained release of drug [20]; AP was also incorporated in sustained-release solid lipospheres intended for use in a suspension formulation and other oral dosage forms [21]. The only study related to AP encapsulation into a nanocarrier for topical application regards the preparation AP-loaded transferosomes topical gel for the alleviation of gout symptoms [22]. To the best of our knowledge, AP has never been encapsulated in a nanoparticle as a strategy to improve skin bioavailability towards a topical application for wound healing management.

Nanostructured lipid carriers (NLCs) are nanometric systems composed by a lipid core with solid and liquid lipids dispersed in an aqueous emulsifier solution [23]. They have provided a great interest for topical application since they are able to protect chemically labile ingredients, improve skin penetration, and modulate drug release [24]. They can also be used in damaged skin, as they are based on non-irritant and non-toxic lipids which can be easily sterilized [16]. NLCs also improve skin hydration, due to their composition and occlusive effect on the skin [25]. When NLCs adhere to the skin, they form a film with an occlusive effect, promoting a decrease in the trans-epidermal water loss and resulting in an increase in water skin content, which facilitates the drug permeation into and through the skin [15].

The main aim of this work was to associate AP with a nanostructured lipid carrier novel formulation, suitable for topical application that could overcome the drug solubility issues and improve the therapeutic index, reducing the side effects. The implications on experimental skin lesions regeneration were addressed.

## 2. Materials and Methods

### 2.1. Materials

Allopurinol (AP) was purchased by Sigma Chemical Co (St. Louis, MO, USA). Precirol® ATO 5 (glyceryl distearate) was provided by Gattefossè (Saint-Priest, France). Myglyol^®^ 812 (caprylic/capric triglyceride) was purchased from Acofarma (Terrassa, Spain). Polysorbate 80 (Tween 80) and acetonitrile were obtained from Merck KGaA (Darmstadt, Germany). 3-(4,5-dimethylthiazol-2-yl)-2,5-diphenyl tetrazolium bromide (MTT) and Dimethyl Sulfoxide (DMSO) were purchased from Sigma–Aldrich (Madrid, Spain). Ultrapure water was provided by reverse osmosis in a MILLI-Q System Elix^®^ 3 from Millipore^®^. All other reagents were of analytical grade, and were used without further purification.

### 2.2. Cell Line and Culture Conditions

A human keratinocyte (HaCaT) cell line was obtained from ATCC (USA). HaCaT cells were grown in RPMI with GlutaMAX™, supplemented with 10% iFBS and 1% penicillin-streptomycin solution at 37 °C in a humidified incubator, with a 5% CO_2_ atmosphere.

### 2.3. Animals

Adult male Wistar rats (280 ± 30 g) were purchased from Instituto de Higiene e Medicina Tropical (Lisbon, Portugal). All animal experiments were conducted according to the animal welfare organization of the Faculty of Pharmacy, Universidade de Lisboa, approved by the competent national authority Direção-Geral de Alimentação e Veteriária (DGAV), and in accordance with the EU Directive (2010/63/EU), the Portuguese laws (DL 113/2013, 2880/2015, 260/2016, and 1/2019), and other relevant legislation.

### 2.4. AP-NLC Preparation

**Figure 1:**
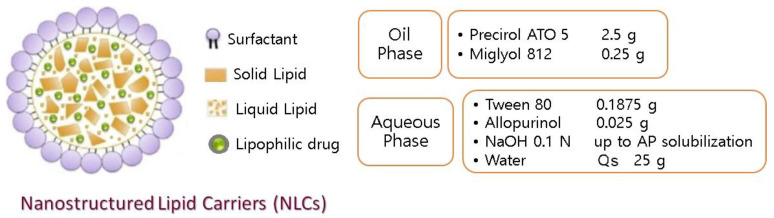
On the left: schematic representation of an individual lipid nanoparticle showing the spatial distribution of the components. On the right: AP-NLC qualitative and quantitative composition. Solid lipid: Precirol ATO 5; liquid lipid: Miglyol 812.

NLC were prepared by an emulsion-sonication method as described elsewhere [26]. The amount of each ingredient of the formulation is shown in Figure 1. In short, 2.5 g of solid lipid (Precirol^®^ ATO 5) and 0.25 g of liquid lipid (Myglyol^®^ 812) were taken in a 50 mL beaker (oil or lipid phase). The aqueous phase consisted of 0.1875 g Tween^®^80, 0.025 g of AP, previously solubilized in NaOH 0.1 N and water up to 25 g. Both the aqueous phase and the lipid phase were heated at 80 °C. After the fusion of the lipids, the aqueous phase was added to an oil phase. The mixture was sonicated with an 18 mm probe (B.BRAUN, 2000 U model) with high power level (output +120; repeating duty cycle: continuous; 10 min). The resulting dispersion was cooled until it reached 25 °C, forming the NLC. Non-loaded particles (empty NLC) were also prepared for control experiments. Three independent batches were prepared.

### 2.5. Size and Zeta Potential

Size and polydispersity index (PdI) were measured by dynamic light scattering (DLS) in a Zetasizer NanoS (Malvern Instruments^®^, Malvern, UK). Measurements were performed at 25 °C, and the scattering information was measured at close to 180°. A value of PdI less than 0.2 indicated a homogenous and monodisperse population. Zeta potential was measured by laser-doppler anemometry in a Zetasizer NanoZ (Malvern Instruments^®^, Malvern, UK). Measurements were performed at 25 °C. Samples were diluted 100 times in water for both size and zeta potential measurements.

### 2.6. Quantification of Allopurinol

The drug content analysis was created through the HPLC method at 254 nm (33 °C) (HPLC Hitachi system LaCrom Elite, Column oven, Diode array detector UV-vis, Pump). The column used was a LiChrospher 100 RP-18 5 μm 125–4. The mobile phase consisted of 0.02 M sodium acetate pH 4.5, adjusted with acetic acid 30% at a flow rate of 1 mL/min (loop: 40 µL).

The total amount of AP was quantified after complete disruption of the NLC with a solvent mixture of methanol/chloroform (1:1 *v*/*v*) (1:10 dilution) and filtration through a 0.2 μm filter. To separate the non-encapsulated AP from loaded NLC, ultrafiltration Amicon^®^ ultracentrifugal filters (molecular weight cut-off, MWCO, 100,000 Da, Millipore, Spain) were used (1500× *g*, 20 min) [27].

The AP encapsulation efficiency (EE) and drug loading (DL) were calculated according to the following equations:

EE (%) = W encapsulated drug/W total drug × 100(1)

DL = W encapsulated drug/W lipid(2)

W total drug: weight of total drug quantified in non-separated samples.

W encapsulated drug: subtraction of the weight of drug quantified in the supernatant of separated samples from the weight of total drug, quantified in non-separated samples.

W lipid: weight of lipids (solid lipid + liquid lipid).

### 2.7. AP-NLC Thermal Analysis

Thermal analysis by DLS (dynamic light scattering) was performed on a Zetasizer Nano S apparatus (Malvern Instruments, Malvern UK), equipped with a Peltier precision temperature control unit (accuracy of 0.1 °C). As a light source, an He-Ne laser with λ = 633 nm was used. The study of the influence of temperature variation in the mean particle diameter was performed between 25 °C and 90 °C (heating phase), and between 90 °C and 25 °C (cooling phase), at a heating/cooling rate of 1 °C/min. The melting point of the solid lipid was 74 °C. The mean diameter of the particles, the PdI, and the total dispersion intensity were measured. AP-NLC and blank samples (10 μL) were previously diluted in 3 mL purified water.

### 2.8. Skin Delivery Studies

In vitro permeation studies were performed using full-thickness skin from newborn pigs as membrane in static-flow Franz diffusion cells. Fresh skin of newborn pigs was obtained from a local abattoir. A suitable size of skin was cut and mounted on the Franz cells. The receptor compartment had a capacity of approximately 4 mL. Prior to permeation study, AP solubility was determined in PBS pH 7.4 containing increasing amount of NaOH 0.1 N in order to select an appropriate receptor fluid complying with sink conditions. PBS: NaOH at 1:4 (*v*/*v*) (pH 12.0) ratio was chosen, since at this ratio, sink condition was achieved. The system was maintained at 32 °C [28]. To ensure such conditions, the receptor phase was immersed in a thermostatic water bath maintained at 32 °C throughout the experiment. Approximately 300 μL of fresh formulation (empty and AP loaded) was spread over the skin. Samples of 500 μL were collected at pre-defined times (2, 4, 6, 8, and 24 h). After every collection, the same volume was replaced with fresh receptor phase maintained at the same temperature. The AP content in the receptor compartment was quantified by HPLC. The data were expressed as the cumulative amount of AP permeated through the skin. To assess the amount of AP retained within the skin, the skin was recovered from the Franz cells, and cleaned with a swab to remove the excess formulation. The amount of AP in the SC was determined using the tape stripping method [29]. The SC was removed by tape-stripping, using 20 adhesive tapes (Scotch 3M, S. Paulo, Brazil). To improve the reproducibility of the tape-stripping technique, a cylinder of 2 kg was used with a pressure of 349.3 g/cm^2^. This pressure was applied for 10 s for each tape. All of the tapes, excluding the first one, were collected in a falcon tube for the extraction process. During the extraction process, 3 mL of methanol were added to the tapes and stirred in a vertical mixer (Ultraturrax, Ultra Turrax T25, IKA, Wilmington, DE, USA). Then, 2 mL MeOH were added and stirred again to complete disruption. Samples were left at room temperature for 30 min, and then centrifuged at 1700 rpm for 10 min rotor (Beckman Optima XL-90, Ultracentrifuge, Palo Alto, CA, USA). The supernatant was recovered to quantify the amount of AP retained in the SC layer. The remaining skin (viable epidermis and dermis layers, E + D) was cut in small pieces, placed in Eppendorf tubes with 1 mL of methanol, and sonicated for 15 min in an ultrasound bath. After 30 min at room temperature, the skin samples were centrifuged at 1700 rpm for 10 min, and the supernatant was recovered to quantify by HPLC the amount of AP retained in the E + D layer.

### 2.9. Rheology Studies

The rheological profile of NLC loaded with AP was evaluated at room temperature (ISO 7884-2) using a Brookfield Rotation Viscosimeter, RV DV-II, SSA, with a spindle 21 (Brookfield Engineering Laboratories, Middleborough, MA, USA). The shear rate [1/s] versus shear stress [Pa] plots (flow curves) were obtained by submitting the samples to a shear rate sweep from 0.61 to 122/s up and down.

### 2.10. Cell-Based Assays

The AP-NLC formulation, free AP, and empty NLC were evaluated in vitro for their cytotoxicity to human cells using HaCaT, an adherent immortalized human keratinocyte non-tumorigenic cell line.

HaCaT cells were harvested by trypsin treatment (TrypLE™) from exponential growth-phase cultures, and transferred to 96-wells plate at a density of 2 × 10^4^ cell/well in 200 µL medium, followed by 24 h incubation for complete cell adhesion, and to reach 60–70% confluence. Cells were treated with different concentrations of AP-NLC, Free AP, and empty NLC serially diluted in RPMI medium. The positive control consisted of HaCaT without the addition of any drug. Stock solution of free AP was prepared with a concentration of 1 mg/mL in NaOH 0.1N diluted to 10 μg/mL in RPMI media. Then, a 2-fold linear dilution ranging from 10 to 0.156 μg/mL in RPMI media was prepared. For the AP-NLC formulation, a suspension containing 100 μg/mL AP was diluted to 10 μg/mL AP in RPMI media, and a 2-fold linear dilution ranging from 10 to 0.156 μg/mL in RPMI media was prepared. For the control NLC formulation (blank), a suspension was prepared in media RPMI, considering the same steps as for the AP-NLC. The plates were incubated for 48 h at 37 °C and 5% CO_2_. After incubation the media was removed, cells were washed with PBS, 50 μL of MTT (0.5 mg/mL) was added to wells, and plates were incubated for 4 h. Then, 200 μL/well of DMSO was added and the plates were shaken for 10–15 min. Cell viability was determined using a microplate reader at 570 nm, by applying the following equation:

(3) $$\mathrm{Cell}\;\mathrm{viability}\;(\%\;\mathrm{of}\;\mathrm{control})=\frac{\mathrm{ABS}\;\mathrm{test}}{\mathrm{ABS}\;\mathrm{control}}\;*\;100$$

where ABS test is the absorbance value obtained for treated cells, and ABS control is the absorbance value obtained for cells incubated with culture medium.

### 2.11. In Vivo Assays

A protocol for the evaluation of the efficacy of the formulation on animal skin lesions based on chemical burns was established using male Wistar rats. The rationale was to reproduce a non-infectious wound model, neither excisional nor incisional, where the SC is defective, and the skin barrier is compromised. After hair removal on the back, rats were treated for five consecutive days with 1 mL of Texapon^®^ N 70 (Sodium Laureth Sulfate, SDS; BASF SE, Ludwigshafen am Rhein, Germany) on an area of 3 × 3 cm^2^, to create lesions on the skin. Before SDS application, animals were lightly anesthetized with isoflurane, the skin was cleaned with a soft cotton pad soaked with lukewarm water and dried with a paper towel, and the skin thickness was measured with a Traceable^®^ Carbon Fiber Calipers, 6″. Skin lesions were scored to establish the evolution of the lesion. To avoid pain, codeine was added to water (30 mg/500 mL). One rat received no SDS application, and was used as a naïve control. The treatment (days 8 to 12) was performed applying 1 mL (1 mg AP) of AP-NLC (3.6 mg AP/kg) on the skin lesion area. On day 8, two animals showing skin lesions were treated with 1 mL of AP-NLC that was applied for five consecutive days on the back skin damaged area. Two animals remained untreated working as the control. At the end of the study, on day 13, rats were sacrificed, and the skin was recovered and fixed in 10% neutral buffered formalin (Sigma-Aldrich, Taufkirchen, Germany) for histological analysis, using standard hematoxylin and eosin staining.

## 3. Results and Discussion

### 3.1. Characterization of AP-NLC Formulation

The selection of NLC components was based on literature reviews and on preliminary assays. The combination of Precirol ATO 5, Miglyol 812 and Tween 80, as the solid lipid, liquid lipid, and surfactant, respectively, has been described for the preparation of drug-loaded NLC for topical application, mainly for lipophilic actives [30]. A suitable solid–liquid lipid combination, which leads to the formation of an appropriate solid nanoparticle matrix, was then chosen and the AP solubility in the lipids at 80 °C was evaluated. It was possible to solubilize the maximum amount of AP in 2.5 g Precirol and 0.25 g Miglyol was 0.025 g. In another preliminary set of experiments, NLC formulations composed of AP in different percentages were prepared to evaluate the effect of AP concentration on the mean size, polydispersity index, and zeta potential of the final particles. AP solubility studies permitted the selection of NaOH as an AP solubility enhancer. Figure 1 shows the composition selected to perform the present study. The AP solubilization approach was also followed in another AP formulation study [18]. Allopurinol has a pKa of 10 (drug molecule has two acidic groups), and, according to the literature, it is in a non-ionized form at low pH [18]. We could only solubilize AP when the pH was increased by the addition of NaOH. The further incorporation of AP into nanoparticles was probably dependent on the degree of drug ionization. Additional tests will be desirable to confirm if AP present in the NLC is mostly in an amorphous state with only few partially crystallized drug molecules, as scarce information exists on allopurinol-to-lipids interaction. According to the literature concerning solid state characterizations of polymer matrix, allopurinol is present as an amorphous material entrapped in polymers, and this condition considerably enhances the dissolution rate of the drug [31]. Recent studies have shown that the use of amorphous actives in topical products responsible for higher saturation solubility creates an increased concentration gradient between the formulation and skin, thus improving the diffusive flux into the skin [32]. In our study, an empty formulation was also prepared to study the characteristics of the formulation without the influence of the drug. The incorporation of AP resulted in an increase of the negative superficial charge of the particles. Based on the macroscopic appearance of the formulations and homogeneity, it was possible to select the formulation containing 0.025 mg AP for further characterization. The pH value of the selected formulation (7.0) is compatible with the skin pH. Skin pH is normally acidic, ranging in pH values of 4–6; however, pH 7 is not considered irritative to the skin [33]. Considering the wound healing purpose, a pH known to improve skin regeneration should be employed. In fact, it was demonstrated that the optimal pH for both keratinocyte and fibroblast proliferation is between pH 7.2 and 8.3 [34].

In the case of drug-carrier skin application, drug permeation into the skin is influenced by the physicochemical characteristics of the particles such as size, morphology, and surface charge being the size and charge the most important factors [35]. The AP-NLC formulation presented a particle diameter of 193 ± 15 nm, PdI of 0.240 ± 0.02, and zeta potential of −61.3 ± 7.0 mV. AP encapsulation efficiency obtained was 52.2%, and the drug loading was 4.74 mg/g. The encapsulation efficiency is comparable to that obtained for other type of nanoparticles [19,20]. Formulation of unloaded particles presented pH 6 and particles presented a particle diameter of 183 ± 6 nm, PdI had a value of 0.243 ± 0.03, and zeta potential was −34.8 ± 1.0 mV. The incorporation of AP into NLC did not particularly influence the characteristics of the formulation, with the exception of the zeta potential. According to the literature, a large positive or negative value of zeta potential of particles indicates good physical stability of nanosuspensions due to electrostatic repulsion of individual particles [36]. According to the obtained results, the incorporation of AP increased the particle electrostatic stability. There is a direct influence of pH on the zeta potential of a colloidal particle. We have checked the ability to reverse the zeta potential of nanoparticles by adjusting the pH, and when the pH decreases, the zeta potential absolute value decreases as well. Although the nanoparticles have been surrounded by a neutral surfactant as Tween, the negatively charged groups created on the surface of the nanoparticle during the formation process are likely to contribute more significantly to the surface potential of the particle [37]. In our case, as above mentioned, the incorporation of AP resulted in an increase of the negative superficial charge of the particles.

Nanoparticles adsorb at the skin surface, creating a film transferring the nanoparticle-loaded drug into the skin, and the smaller the size, the better percutaneous penetration [38]. The particle diameter obtained is adequate to create a film of lipid particles and to promote skin delivery [39]. Considering the application in wounds, NLC are capable of increasing residence time thus reducing the healing time process [39].

**Figure 2:**
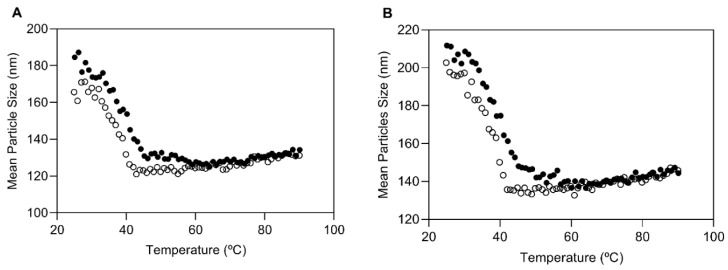
Thermal analysis of naïve NLC (**A**) and AP-loaded NLC (**B**): closed circles from 25 °C to 90 °C and open circles from 90 °C to 25 °C.

The effect of temperature on the particle size of NLC formulations was assessed using DLS. This analysis is often used to study the physical behavior of solid lipid nanoparticles suspensions during processes that include heating and cooling variations as a stress test of physical stability [40]. Empty NLC particles and AP-loaded NLC particles exhibited the same behavior due to the same lipid amount present in the tested formulations. A decrease in the size, expressed in nm, occurred around the temperature of 40–45 °C, for both formulations during the heating process, far from the temperature of solid lipid melting (~74 °C). The opposite behavior was observed during the cooling process, where an increase in the size of both formulations occurred at approximately the same temperature. These results are presented in Figure 2. NLC showed high stability within different temperature conditions as observed for other lipid nanoparticles [41]. The influence of the temperature variation in the ranges 25–90 °C and 90–25 °C on particle size was independent of the presence of AP. During the heating phase, a reorganization of the lipid matrix might have occurred, considering the size changes; however, the particles were able to recover their size which is indicative of formulation stability at high temperatures.

The rheology assay is important to understand the spreadability and flow behavior of the formulation, which is a significant aspect for topical administration to assure patients compliance. Viscosity was measured for AP-loaded and empty NLC. Semisolid formulations show advantages for topical application, as they prolong the contact time with the skin, reducing the number of applications.

**Figure 3:**
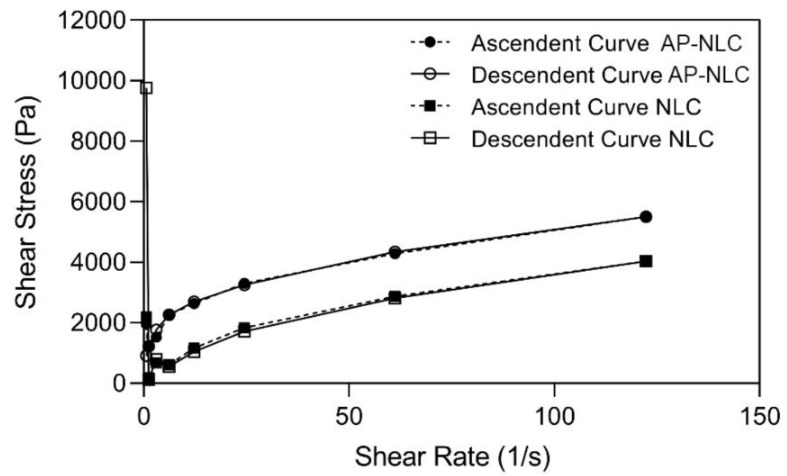
Rheological analysis of empty and AP-loaded NLC. Circles: AP-NLC, Squares: NLC. Closed symbols: Ascendent curve; Open symbols: Descendent curve.

As shown in Figure 3, in rheological terms, both formulations present pseudoplastic non Newtonian behavior. By increasing the shear rate, the viscosity decreases, but the formulation easily recovers its structure after removal of applied force, with no hysteresis. On the other hand, the incorporation of AP increases the viscosity value when compared to empty NLC at the same shear rates. In general, the increase in viscosity is due to an increase in the volume of lipid phase [25]. In our study, being that the volume of lipid phase is the same for loaded and non loaded NLC, the difference in zeta potential may have played a role in viscosity, being that the loaded nanoparticles are strongly anionic (<−30 mV) in comparison with non-loaded ones. In any case, both formulations present a rheological profile adequate for topical application.

### 3.2. Skin Delivery Study

**Figure 4:**
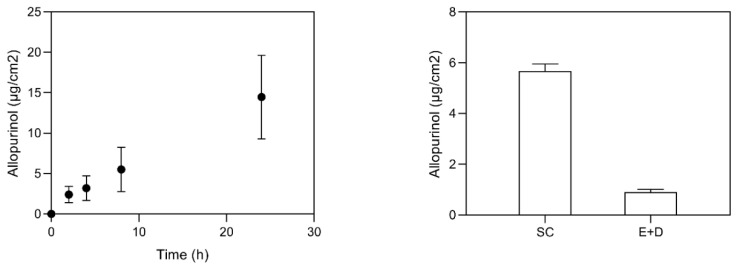
Permeation (**left**) and retention (**right**) assays for AP-NLC formulation. The results are expressed as mean ± S.D. (*n* = 6).

The measurement of drug skin permeation from a given dosage form is an important assay for the development of a new topical formulation [42]. The most common method employs an open chamber design, and can be used with a synthetic membrane, a tissue construct, or a biological sample, such as skin [42]. Permeation and retention studies were performed using Franz diffusion cells. Permeation studies allow understanding the formulation behavior when in contact with the skin, thus predicting drug skin permeation in vivo, and possible systemic effect [29]. The skin barrier provides a hydrophobic environment necessary for preventing and controlling drug delivery. In vitro permeation studies were essential to evaluate the ability of AP-NLC to penetrate through the skin layers, instead of being retained at the skin surface. Just as important as drug permeation results is the skin drug retention in order to enlarge the period of drug contact with the skin. Permeation and retention of AP-NLC were assessed using newborn pig skin as a human skin surrogate. Figure 4 shows the results of permeation and skin retention obtained in different skin layers when the drug is loaded in the NLC system.

The AP retention was higher in the SC compared to the other skin layers. It seems that the encapsulation of the drug in the developed formulation facilitates the skin deposition as carrier association may increase drug solubility and partition in the skin. Due to the high lipophilicity, AP should interact mostly with the stratum corneum components. Therefore, it is expected to find AP in the skin and, to a greater extent, in the stratum corneum. AP from AP-loaded NLC was able to cross the skin, and reached the receptor fluid (Figure 4, left). The permeation and retention assays for free-AP (in solution) were hampered by its solubility, and no detectable AP was found in the receptor phase or within the skin. Comparison with data from the literature is difficult as the only study reported so far presents the cumulative AP permeation through rat skin from transfersomal gel in percentage values [22].

### 3.3. Assessment of Cellular Cytotoxicity against HaCaT Cells

**Figure 5:**
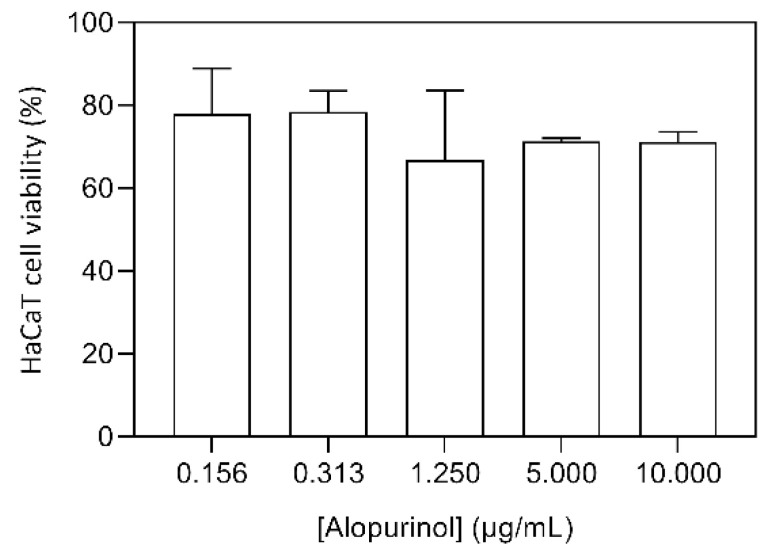
Evaluation of AP loaded NLC cytotoxicity on the HaCaT cell line. Results are expressed as mean ± standard error of a representative experiment.

A desirable feature of a compound for the topical application is the absence of cytotoxicity against skin cells. HaCaT cell line has been widely used as a reproducible model for the characterization of human skin keratinocytes. Therefore, a HaCaT keratinocyte cell line was used to assess AP-NLC formulation cytotoxicity in mammalian cells, using the MTT cell viability assay. AP-loaded NLC were tested and presented no cytotoxicity to HaCaT cells on the range of concentrations tested (between 0.156 and 10,000 µg/mL) (Figure 5). The empty formulation (vehicle) and free AP were also tested for the same concentrations, and presented no toxicity. Overall, the results obtained indicate the safety of the developed formulation.

### 3.4. In Vivo Assay

In this preliminary assay, a non-infectious model based on the formation of skin lesions was developed on rat skin, in order to simulate a skin barrier damage, and to evaluate AP ability to regenerate damaged skin. We decided to avoid an excisional model, since rodent excisional wounds heal by contraction, while humans heal by re-epithelialization [43]. For the purpose, before the administration of SDS gel responsible for the lesion formation, skin thickness was measured daily.

**Figure 6:**
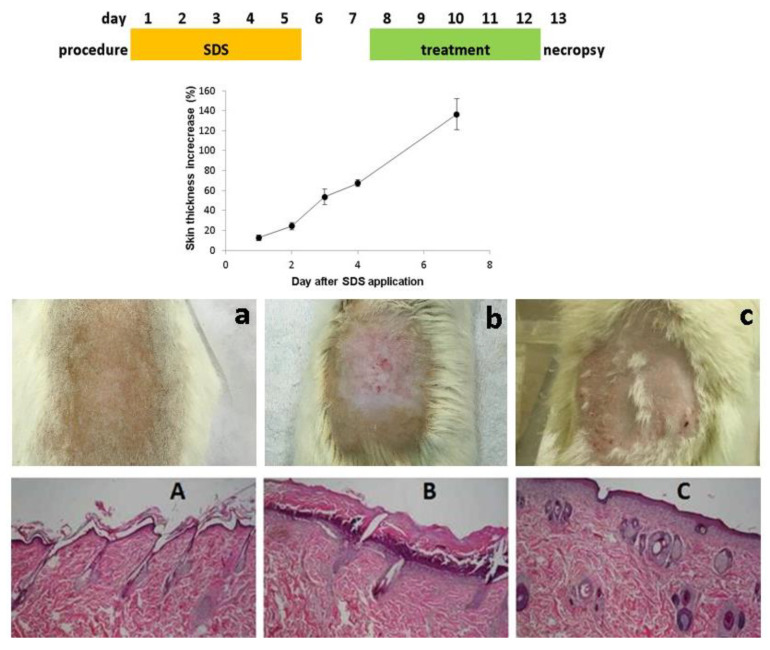
Top: timeline representation of the in vivo model; in the middle: skin thickness (%) during the application of the SDS gel. Bottom: photographs of: (**a**) the skin from naïve control rat skin; (**b**) rat skin treated topically with SDS gel; (**c**) rat skin treated topically with SDS gel followed by topical application of AP-NLC; representative microphotographs of skin stained with haematoxylin and eosin (200× magnification) from: (**A**) naïve control rat skin; (**B**) rat skin treated topically with SDS gel; (**C**) rat skin treated topically with SDS gel followed by topical application of AP-NLC.

The skin thickness remarkably increased during the application of the SDS gel (Figure 6). In the case of skin and soft tissue inflammation and/or infection, it is common to observe an increase of skin thickness as a consequence of microbial skin invasion and blood cells infiltration. The application of AP-loaded NLC resulted in a decrease of skin lesion, when compared to non-treated controls. Regarding any eventual toxicity signs, on average, the animal body weight loss was not higher than 6% during the first 8 days, and the animals recovered their weight at the end of the experiment. Animals presented no behavioral changes during the experiment time course. Macroscopic lesions observations (Figure 6a–c) were corroborated by the histological analysis (Figure 6A–C). A similar non-infectious wound model was carried out in mice, to test the efficacy of topical insulin nanoparticles [1]. Results were also favorable regarding tissue regeneration after skin burn, based on macroscopical analysis over five days of treatment [1].

Histology analysis showed that the application of 40% SDS gel on rat skin for five consecutive days resulted in extensive and severe dermatitis lesions with epidermal necrosis (Figure 6B). Skin treated with AP-NLC presenting macroscopic signs of healing (Figure 6C) showed microscopically advanced healing of dermatitis lesions (Figure 6C). These results were encouraging to proceed with a complete in vivo assay to test the potential of AP formulation in the presence of tissue damage and excessive host immune response, and compare it with several controls. Despite occlusion being generally accepted as preventing wound desiccation, which may stimulate epidermal cell migration, a study with a NLC composition very similar to the one presented in our work [44] demonstrated the lack of wound healing activity of empty carriers. It would be interesting to determine the severity of associated inflammatory skin disease that greatly contribute to change drug penetration profile. In the study from Madigan et al., the topical application of AP (direct application of 30 µg/wound) resulted in a reduction of ROS production and significant delay in wound closure [8]. In our study using another wound healing model and applying 1 mg AP to each wound, we were able to achieve a reduction of skin lesion in comparison to non-treated controls. Skin lesion drug targeting with a topical AP formulation thus appears as a promising strategy to control wounds. Kimball and co-workers have created wounds in diet-induced obese mice and injected animals with AP [45]. Injections started on day three, and resulted in a significant improvement in healing through day seven, suggesting that xanthine oxidase antagonists may represent a target for the remission of inflammation in diabetic wounds. These encouraging results were obtained using a parenteral approach to treat wounds. In our work, with the administration of AP locally to wound sites we made a step forward on the understanding how AP blocking uric acid could help wound healing.

## 4. Conclusions

In this work, NLCs were chosen as systems with the ability to solubilize, incorporate, and deliver AP into the skin. A NLC formulation was prepared and characterized. The formulation presented particles with suitable nanometric size, high drug incorporation, and a zeta potential value to assure a good electrostatic stabilization. The rheological behavior showed a prolonged contact between the drug and the site of action, and even the in vitro skin retention and penetration showed good results for the topical administration of AP with no evidence of toxicity towards skin cells. According to the results obtained in this work, it is possible to conclude that the formulation shows potential for the topical AP delivery. Despite being initial studies, in vivo assay proved that AP-NLC formulation has potential to be used in the regeneration of skin lesions.

## Data Availability

The data presented in this study are only available in this article.
